# The safety of PolyGlycopleX^® ^(PGX^®^) as shown in a 90-day rodent feeding study

**DOI:** 10.1186/1475-2891-8-1

**Published:** 2009-01-16

**Authors:** Ray A Matulka, Michael R Lyon, Simon Wood, Palma Ann Marone, Daniel J Merkel, George A Burdock

**Affiliations:** 1 Burdock Group, 801 North Orange Avenue, Suite 710, Orlando, FL 32801 USA; 2 Canadian Centre for Functional Medicine, 1552 United Boulevard, Coquitlam, BC, V3K 6Y2, Canada; 3 University of British Columbia, Food, Nutrition and Health Program, 2357 Main Mall, Vancouver, B.C., V6T 1Z4, Canada; 4 Eurofins|Product Safety Laboratories, 2394 Highway 130, Dayton, NJ 08810 USA

## Abstract

**Background:**

This study was designed to evaluate the safety of PolyGlycopleX^® ^(PGX^®^), a novel viscous dietary polysaccharide (fiber), when administered to Sprague Dawley^® ^rats in the diet for 90 days.

**Methods:**

Groups of ten male and ten female rats each consumed PGX mixed in the diet at levels of 0, 1.25, 2.5 or 5.0% for 90 days, then evaluated for toxicological effects on parameters that included neuromotor activity, body weight, clinical chemistry, urinalysis, hematology, and histopathology.

**Results:**

Mean body weight, mean feed consumption and food efficiency in the treated groups were generally comparable to controls for both male and female rats. No changes were noted in neuromotor behavior, and histopathological analysis revealed no significant changes between treated and control animals. There were no differences in mean organ weight, organ-to-body weight or organ-to-brain weight values between controls and treated animals. Decreased red blood cell count occurred in the high dose males and increases in aspartate and alanine aminotransferase enzyme levels and triglycerides, while significant decreases in serum sodium, potassium and chloride concentrations were observed in the females fed 5.0% PGX. However, the decreased mineral concentrations may be the result of significantly increased urinary volume in both males and females at the high dose, with a concomitant decrease in urinary specific gravity (males and females) and protein concentration (females). These results were within historical control values, did not correlate with any histopathological changes, and were not considered adverse.

**Conclusion:**

The results indicate a no observed adverse effect level (NOAEL) for PGX at 5.0% of the diet, corresponding to an average daily intake of 3219 and 3799 mg/kg bw/day in male and female rats, respectively.

## Background

Dietary fiber has been declared a nutrient under the Nutrition Labeling and Education Act of 1990, for the purposes of nutrition labelling. The Institute of Medicine [1] states that "Dietary fiber consists of nondigestible food plant carbohydrates and lignin in which the plant matrix is largely intact. Nondigestible means that the material is not digested and absorbed in the human small intestine." Dietary fibers are defined as either soluble or insoluble (depending on solubility characteristics), and fermentable or non-fermentable, depending on the ability of anaerobic bacteria in the colon to digest the fiber. This definition encompasses a broad category of carbohydrates, including cellulosic plant material, pectins, gums, and non-plant (*e.g.*, animal or fungal) dietary substances. Many positive benefits have been associated with diets rich in fiber, including a reduced risk of diabetes and cardiovascular disease, reduced risk of hypertension, as well as a reduction of total and low density lipoprotein (LDL) cholesterol [2] and an increase in high density lipoprotein (HDL) cholesterol [3-6]. The type of fiber may affect food consumption, as some fibers have the ability to swell and hold water, inducing a feeling of fullness [7], which can aid in the maintenance of, or a reduction in, body weight.

In the United States, most people consume less fiber than the daily value recommendation of 25 g. Food manufacturers have started evaluating the combination of specific fiber types to produce a complex fiber food ingredient that may provide the positive benefits of fiber, without compromising on taste or texture. PGX is a proprietary product manufactured from glucomannan (prepared from konjac powder), sodium alginate, and xanthan gum. Glucomannan, a β-D-(1-4)-linked linear polymer of glucose and mannose substituted with O-acetate every 9 to 19 sugar units [8], produces a viscous fiber that is highly fermentable in the large intestine. Alginates are algal polysaccharides that are used in the food industry as stabilizers or thickening agents. Xanthan gum, a polysaccharide produced by the bacterium *Xanthomonas campestris*, has a viscous property that adds bulk and increases stool water retention. Both the gum and the alginate are also considered viscous, indigestible dietary fibers, shown to lower the rate of small intestinal absorption of metabolizable nutrients and may potentially decrease caloric intake from the small intestine [9-11]. Although each of these fibers has been studied for their safety when used alone or with other fibers, this unique product, formed from the combination of fibers, has not been evaluated. Therefore, this study was designed to assess the safety of subchronic administration of PGX to male and female Sprague Dawley^® ^rats.

## Methods

The study was conducted at Eurofins|Product Safety Laboratories (Dayton, NJ) under the sponsorship of InovoBiologic, Inc. (Calgary, Alberta).

### Test Article

The composition of the test article is that of a granular powder produced and marketed under the trade name PGX^® ^(InovoBiologic, Inc., Calgary, Alberta), an off-white powder soluble in water, that has been manufactured from glucomannan, xanthan gum and sodium alginate by a proprietary process. PGX was added to PMI LabDiet^® ^Certified Rodent Meal #5002 and mixed for 20 minutes at room temperature to form a homogeneous mixture. The vehicle diet was mixed under the same conditions. The diets were prepared once per week and refrigerated until use. The dietary concentration was 0, 12,500, 25,000, and 50,000 ppm, corresponding to 0, 1.25, 2.5, and 5.0% in the diet.

### Animals

Sprague Dawley^® ^rats (ten/sex/group) were supplied from Harlan, Inc. (Indianapolis, IN), and were seven to eight weeks of age at the beginning of the study. The animals were housed singly in an animal room with a 12-hour light/dark cycle at a temperature and relative humidity range of 22 ± 3°C and 30–70%, respectively. The animals were acclimated for at least five days prior to testing. PMI LabDiet^® ^Certified Rodent Diet Meal #5002 was available *ad libitum *during the acclimation period. Water was available *ad libitum *throughout the study.

### Study Design

This study was conducted based on OECD Guidelines for Testing of Chemicals, Section 4 (Part 408): Health Effects (1987) and US FDA Redbook 2000, IV.C.3a. "Short-term toxicity studies with rodents" (2003). It also complied with OECD Principles of Good Laboratory Practices [12] and US FDA Good Laboratory Practices [13]. All work undertaken by the testing laboratory was in accordance with the most recent *Guide for the Care and Use of Laboratory Animals *[14], and the laboratory received accreditation from the Council on Accreditation of the Association for Assessment and Accreditation of Laboratory Animal Care (AAALAC International) and approval from the National Institutes of Health Office of Laboratory Animal Welfare (OLAW) for animal experimentation. Briefly, four groups of twenty rats (ten/sex/group) were each provided a diet containing 0, (Group 1), 12,500 ppm (1.25%, Group 2), 25,000 ppm (2.5%, Group 3), or 50,000 ppm (5%, Group 4) PGX for 92 days (13 weeks). The animals were observed daily for viability, behavioral changes and signs of gross toxicity, and weekly for a battery of detailed observations. Individual food consumption was recorded weekly. Mean daily food consumption was calculated for each sex/dietary level at each week and for the overall (Days 1 to 92) testing interval. The Group mean daily intake of test article was calculated by multiplying the Group mean daily food consumption (grams) for a given interval by dose concentration and then dividing by the Group mean body weight (grams) for a given interval. Mean daily food efficiency was also calculated for each sex/dietary level based on body weight gain and daily food consumption data. Body weights were recorded two times during the acclimation period, prior to test initiation (Day 1), weekly during the study, and just prior to terminal sacrifice. Mean daily body weight gains were calculated for each sex/dietary level at each interval and for the overall testing interval (Days 1–94). A Functional Observational Battery (FOB) and a Motor Activity (MA) test were performed on all animals during Week 12 of the study. Included in this evaluation were observations while the rat was in the home cage, as well as open field observations. These observations included both forelimb and hindlimb grip strength, as well as hindlimb foot splay. A clinical pathology analysis was conducted on samples collected on test Day 87 for hematology, clinical chemistry, and urinalysis, and on Days 93 (males) and 94 (females) for coagulation.

Animals were fasted for at least 15 hours and then placed in metabolism cages one day before clinical pathology evaluation. Urine was collected from each animal and analyzed for quality, pH, ketone, color, glucose, bilirubin, clarity, specific gravity, blood, volume, protein, urobilinogen and microscopic urine sediment. Urine protein was measured on an Olympus^® ^AU640 clinical chemistry analyzer. Other urine constituents were semi-quantitatively measured on a Bayer Clinitek^® ^Atlas™ Automated Urine Chemistry analyzer (Siemens Medical Solutions Diagnostics, Tarrytown, NY). Sediments from all urine specimens were evaluated microscopically.

Animals were fasted overnight and blood samples were collected for hematology (except prothrombin and partial thromboplastin time) and clinical chemistry *via *orbital sinus bleeding under isoflurane anesthesia on Day 87 of the study. Blood samples for determination of prothrombin time and activated partial thromboplastin time were taken *via *the inferior vena cava under isoflurane anesthesia prior to terminal sacrifice on Day 93 (males) or Day 94 (females).

Hematology parameters included erythrocyte count, hemoglobin concentration, hematocrit, mean corpuscular volume, mean corpuscular hemoglobin, red cell distribution width, absolute reticulocyte count, platelet count, total white blood cell and differential leukocyte count. Mean corpuscular hemoglobin concentration was also calculated. Blood smears were prepared and evaluated. Complete blood counts, including reticulocytes, were determined on a Bayer^® ^Advia 120 hematology analyzer (Diamond Diagnostics, Holliston, MA) or determined from microscopic evaluation of the blood smear. Wright-Giemsa-stained blood smears from all animals were examined microscopically for confirmation of automated results and evaluation of cellular morphology. Coagulation times were determined on a Sysmex^® ^CA-1000 Coagulation Analyzer (Sysmex America, Inc., Mundelein, IL).

Clinical biochemistry values analyzed were: serum aspartate aminotransferase, serum alanine aminotransferase, sorbitol dehydrogenase, alkaline phosphatase, total bilirubin, blood urea nitrogen, blood creatinine, total cholesterol, triglycerides, fasting glucose, total serum protein, albumin, globulin, calcium, inorganic phosphorus, sodium, potassium, and chloride. Serum clinical chemistry parameters were determined on an Olympus^® ^AU640 clinical chemistry analyzer (Center Valley, PA). Serum samples from two randomly chosen animals were pooled and a viral screen was performed.

Gross necropsies were performed on all study rats at study termination, and selected organs and tissues from the control (Group 1) and high dose (Group 4) groups were harvested, weighed, and evaluated for histological changes (Table 1) following staining with hematoxylin and eosin and examination by light microscopy.

**Table 1:** Tissues collected at necropsy and microscopically evaluated in the control and high dose treatment groups

Lungs	Trachea	Thymus	Heart
Sternum with bone marrow	Adrenals	Liver	Spleen
Kidneys	Thyroid/parathyroid	Urinary bladder	Ovaries uterus & fallopian tubes
Vagina	Esophagus	Ileum	Cecum
Peripheral nerve (sciatic)	Jejunum	Stomach	Duodenum
Accessory genital organs (prostate and seminal vesicles)	Colon	Rectum	Representative lymph node (mesenteric & mandibular)
Pancreas	Pituitary	Aorta	Female mammary gland
Harderian gland	Skin	Nasal turbinates	Skeletal muscle
Epididymides	Testes	Eyes	

In addition, gross lesions of potential toxicological significance noted in any test groups at time of euthanasia were also examined. These included the uterus and fallopian tubes from two low dose and one intermediate dose female(s) and the testes from one intermediate dose male. Relative organ weights (g/kg body weight) were calculated using terminal body weights.

### Statistical Analysis

Statistical analysis of the in-life and organ weight data [*i.e*., means and standard deviations of body weight, daily body weight gain, daily food consumption, daily food efficiency, organ weight, and organ-to-body/brain weight ratio, and the functional observation battery (FOB)] were initially evaluated for variance homogeneity and normality by Bartlett's test. When the variances were homogeneous, treated and control groups were compared using a One-Way Analysis of Variance (ANOVA), followed by comparison of the treated groups to control by Dunnett's t-test for multiple comparisons. Otherwise, groups were compared using a non-parametric method (Kruskal-Wallis non parametric analysis of variance followed by Dunn's test). Motor activity data was analyzed by Two-Way repeated measures ANOVA. Means and standard deviations were calculated for the quantitative clinical pathology data using Levene's test, and the Shapiro-Wilk test for normality. If variances were not significantly different, groups were compared using a One-Way Analysis of Variance (ANOVA) followed by Dunnett's t-test for multiple comparisons. If the Shapiro-Wilk test was not significant but Levene's test was significant, a robust version of Dunnett's test was utilized. When Levene's test indicated significantly different variances, groups were compared using a non-parametric method (Kruskal-Wallis non parametric analysis of variance) followed by Dunn's test. Male and female rats were evaluated separately, and differences among groups were judged to be significant at a probability value of *P *≤ 0.05.

## Results

**Figure 1:**
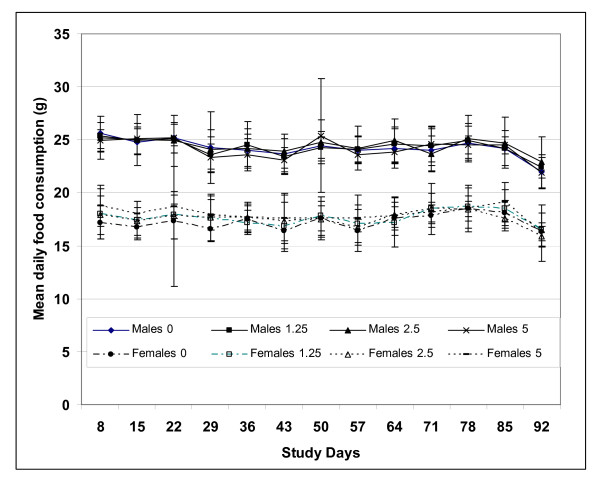
**Food consumption for male and female rats exposed for 90 days to PGX^®^**. Values represent mean ± SD of 10 rats/sex/group.

The mean overall daily intake in male rats fed dietary concentrations of 0, 12,500, 25,000, and 50,000 ppm PGX (equivalent to 0, 1.25, 2.5, and 5.0% of the diet, respectively) during this study was approximately 0, 806, 1617, and 3219 mg/kg bw/day, respectively. For the same dietary concentrations, female rats consumed a mean overall daily intake of approximately 0, 918, 1828, and 3799 mg/kg/day PGX, respectively. Mean daily food consumption and food efficiency for all groups of male and female rats that consumed 1.25, 2.5, or 5.0% PGX were comparable with control values (Figure 1).

There were no mortalities during the study and all animals appeared active and healthy during the course of the study. Transient clinical signs included intermittent and sporadic light brown feces for one intermediate group female (Day 57), all high dose males (Days 57 and 64) and 2/10 and 6/10 high dose females (Days 57 and 64, respectively). These findings were considered non adverse as all animals recovered by the end of the study. One high dose male was found emaciated on Day 57, the result of a faulty water valve, unrelated to test substance administration and data from this animal was removed from the group averages for Days 57 and 64 for body weight and for Days 57, 64, and 1–92 (overall) for parameters of body weight gain, food consumption, food efficiency and mean daily intake. One high dose female was noted with emaciation and a reduced fecal volume during the detailed clinical observations on Day 92. This animal had a reduced body weight, body weight gain, food consumption and feed efficiency during study Days 84–92. A noted reduction in absolute and relative thymus weight (necropsy Day 94) was confirmed histologically as slight atrophy, however, there were no clinical pathology findings associated with this animal and the cause for emaciation remains undetermined pathologically. Due to their short duration, limited severity and singular appearance in one animal within the group, these findings were considered incidental and not test article-related.

**Figure 2:**
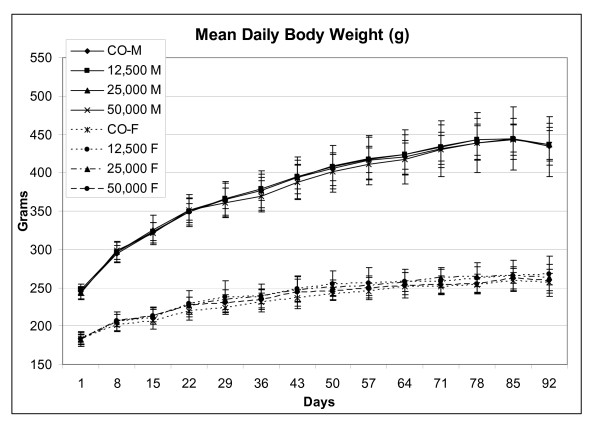
**Mean daily body weight (g) of male and female rats administered PGX^®^**.

Weekly mean body weight and body weight gain for all groups that consumed PGX were comparable to control values (Figure 2). Both eyes of all the study rats were examined by focal illumination and indirect ophthalmoscopy prior to study initiation and on Day 88 of the study; no treatment related effects were noted. The Functional Observational Battery and Motor Activity tests conducted in both the male and female rats that consumed PGX were comparable to results from the control rats.

Male rats fed 5% PGX in the diet had a significant decrease in red blood cell count at the end of the study (*P *< 0.05), but this did not follow a dose-response effect and there were no statistically significant effects in any other red cell or coagulation parameters, and no hematological effects in the female treatment groups. Analysis of the mean clinical chemistry parameters found that male rats fed 5% PGX in the diet showed a significant increase in the serum albumin concentration (*P *≤ 0.05); however, no other effects were noted in any of the remaining clinical chemistry parameters evaluated in the male rats at any PGX treatment level (Table 2). In the female rats fed PGX at 5% of the diet, significant increases were noted in mean aspartate aminotransferase, alanine aminotransferase, and serum triglyceride concentrations (Table 3). A significant decrease in the inorganic phosphorous concentration was found in the female rats fed 2.5% PGX, but not in the 5.0% or 1.25% dose groups. In female rats fed 5.0% PGX, there were significant decreases in serum sodium, potassium, and chloride levels (*P *≤ 0.05). Serological study showed no detectable titers against the pathogens and antigens tested.

**Table 2:** Mean clinical biochemistry values of male rats administered PGX^® ^for 90 days

Parameter (units)	Control	1.25%	2.5%	5.0%
Aspartate Aminotransferase (U/L)	107 ± 32	82 ± 7	85 ± 15	92 ± 13
Alanine Aminotransferase (U/L)	50 ± 16	43 ± 7	46 ± 9	48 ± 7
Sorbitol Dehydrogenase (U/L)	8.4 ± 2.7	8.3 ± 3.0	8.2 ± 2.0	8.3 ± 2.0
Alkaline Phosphatase (U/L)	107 ± 30	112 ± 32	96 ± 13	113 ± 25
Total Bilirubin (mg/dL)	0.12 ± 0.01	0.12 ± 0.01	0.12 ± 0.02	0.13 ± 0.02
Blood Urea Nitrogen (mg/dL)	17 ± 2	18 ± 2	16 ± 1	17 ± 2
Creatinine (mg/dL)	0.31 ± 0.04	0.32 ± 0.03	0.32 ± 0.03	0.33 ± 0.03
Cholesterol (mg/dL)	99 ± 19	102 ± 25	100 ± 15	92 ± 11
Triglycerides (mg/dL)	36 ± 11	45 ± 19	44 ± 16	44 ± 10
Glucose (mg/dL)	115 ± 12	113 ± 5	113 ± 8	109 ± 8
Total Protein (g/dL)	6.2 ± 0.2	6.2 ± 0.2	6.2 ± 0.3	6.2 ± 0.2
Albumin (g/dL)	3.1 ± 0.2	3.1 ± 0.1	3.2 ± 0.1	3.3 ± 0.1^a^
Globulin (g/dL)	3.1 ± 0.2	3.0 ± 0.2	3.0 ± 0.2	2.9 ± 0.2
Calcium (mg/dL)	10.3 ± 0.3	10.3 ± 0.3	10.4 ± 0.3	10.7 ± 0.3
Inorganic Phosphorus (mg/dL)	6.9 ± 0.4	7.0 ± 0.7	7.1 ± 0.6	7.5 ± 1.0
Sodium (mmol/L)	144.2 ± 5.6	145.5 ± 4.8	145.0 ± 4.8	145.6 ± 6.2
Potassium (mmol/L)	5.39 ± 0.28	5.39 ± 0.4	5.44 ± 0.23	5.34 ± 0.46
Chloride (mmol/L)	102.4 ± 4.0	103.4 ± 3.3	102.9 ± 2.9	102.9 ± 4.1

^a^Statistically different from control (P ≤ 0.05) by Dunn's test

**Table 3:** Mean clinical biochemistry values of female rats administered PGX^® ^for 90 days

Parameter (units)	Control	1.25%	2.5%	5.0%
Aspartate Aminotransferase (U/L)	84 ± 13	86 ± 6	88 ± 10	101 ± 18^a^
Alanine Aminotransferase (U/L)	32 ± 3	38 ± 4	40 ± 7	54 ± 14^a^
Sorbitol Dehydrogenase (U/L)	10.0 ± 2.0	9.2 ± 1.7	10.3 ± 2.2	10.4 ± 2.0
Alkaline Phosphatase (U/L)	87 ± 31	97 ± 18	89 ± 25	103 ± 24
Total Bilirubin (mg/dL)	0.16 ± 0.03	0.16 ± 0.02	0.16 ± 0.02	0.18 ± 0.02
Blood Urea Nitrogen (mg/dL)	18 ± 2	17 ± 2	19 ± 1	18 ± 2
Creatinine (mg/dL)	0.41 ± 0.03	0.42 ± 0.04	0.44 ± 0.03	0.43 ± 0.04
Cholesterol (mg/dL)	98 ± 8	106 ± 11	102 ± 16	111 ± 13
Triglycerides (mg/dL)	25 ± 5	31 ± 8	29 ± 8	34 ± 8^a^
Glucose (mg/dL)	115 ± 8	111 ± 7	115 ± 10	111 ± 7
Total Protein (g/dL)	6.5 ± 0.3	6.5 ± 0.2	6.5 ± 0.3	6.4 ± 0.3
Albumin (g/dL)	3.5 ± 0.2	3.5 ± 0.2	3.5 ± 0.2	3.5 ± 0.2
Globulin (g/dL)	3.0 ± 0.2	3.0 ± 0.2	3.1 ± 0.2	2.9 ± 0.2
Calcium (mg/dL)	10.7 ± 0.4	10.6 ± 0.3	10.5 ± 0.1	10.7 ± 0.4
Inorganic Phosphorus (mg/dL)	5.9 ± 0.5	5.5 ± 0.6	4.9 ± 0.4^b^	5.4 ± 0.7
Sodium (mmol/L)	143.5 ± 5.8	141.0 ± 6.9	143.4 ± 5.6	136.6 ± 5.1^b^
Potassium (mmol/L)	5.02. ± 0.28	4.88 ± 0.25	4.89 ± 0.25	4.71 ± 0.25^b^
Chloride (mmol/L)	104.9 ± 4.0	102.3 ± 5.1	104.8 ± 3.4	100.4 ± 3.^b^

^a ^Statistically different from control (*P *≤ 0.05) by Dunn's test; ^b ^Statistically different from control (*P*≤ 0.05) by Dunnett test

Evaluation of urinalysis parameters found that urinary volume was increased in both males and females fed 5.0% PGX, while the specific gravity of the urine was decreased at the highest dose level in both male and female rats (Table 4). The protein content in the urine of female rats administered 5.0% PGX in the diet was significantly decreased compared to control animals. Urinary pH was increased in male rats fed 2.5 and 5.0% PGX, and in female rats fed 5.0% PGX.

**Table 4:** Mean ± SD^c ^of urinalysis values of male and female rats administered PGX^® ^for 90 days

Parameter (units)	Control	1.25%	2.5%	5.0%
Males				
Volume (mL)	3.0 ± 1.5	4.0 ± 1.7	6.3 ± 4.6	6.4 ± 3.3^a^
Specific Gravity	1.074 ± 0.02	1.060 ± 0.017	1.054 ± 0.025	1.045 ± 0.018^b^
pH	6.2 ± 0.3	6.4 ± 0.3	6.6 ± 0.3^b^	7.1 ± 0.5^b^
Urobilinogen (EU/dL)	0.6 ± 0.4	0.4 ± 0.4	0.4 ± 0.3	0.5 ± 0.4
Total Protein (mg/dL)	876+683	808 ± 279	745 ± 412	457 ± 346

Females				
Volume (mL)	2.9 ± 3.3	4.0 ± 2.7	1.0 ± 1.0	6.8 ± 2.9^a^
Specific Gravity	1.055 ± 0.026	1.042 ± 0.017	1.070 ± 0.011	1.027 ± 0.008^b^
pH	6.0 ± 0.0	6.2 ± 0.4	6.1 ± 0.2	6.9 ± 0.2^a^
Urobilinogen (EU/dL)	0.3 ± 0.3	0.3 ± 0.3	0.4 ± 0.4	0.2 ± 0.0
Total Protein (mg/dL)	258 ± 245	196 ± 374	111 ± 66	61 ± 46^a^

^a^*P *≤ 0.05 by Dunn's test; ^b^*P *≤ 0.05 by Dunnett/Tamhane-Dunnett test; ^c^SD = standard deviation

At study termination the rats were euthanized, organs removed, weighed, and histopathological analysis was conducted. No gross abnormalities attributable to PGX administration were found. Incidental findings included fluid-filled uteri in some of the females in all groups (one rat each in control and 2.5% treatment group, two rats each in the 1.25 and 5.0% treatment groups, respectively), and a light brown encapsulated firm mass, anteriomedial of the right testis in one male fed 2.5% PGX, for which there were no histological correlate. There were no differences between the control group and any test group in mean organ weight (Table 5), as well as organ-to-body weight and organ-to-brain weight (Table 6) in either males or females. Histopathological analysis found that all microscopic changes observed in the animals sacrificed at study termination were of a type commonly observed in control laboratory rats, and were of comparable incidence in controls and high dose groups in this study.

**Table 5:** Mean organ weights of male and female rats that consumed PGX^® ^for 90 days

Organ	Control	1.25%	2.5%	5.0%
Males				

Brain	1.98 ± 0.12	1.97 ± 0.06	2.00 ± 0.12	1.99 ± 0.10
Heart	1.42 ± 0.13	1.46 ± 0.09	1.41 ± 0.11	1.43 ± 0.10
Kidneys (paired)	3.09 ± 0.23	2.90 ± 0.20	2.91 ± 0.23	2.89 ± 0.21
Liver	11.11 ± 1.10	11.12 ± 0.89	11.62 ± 1.29	11.76 ± 0.68
Spleen	0.81 ± 0.15	0.76 ± 0.08	0.75 ± 0.07	0.81 ± 0.07
Testes (paired)	4.03 ± 0.32	4.04 ± 0.27	4.01 ± 0.25	4.08 ± 0.19
Adrenals (paired)	0.063 ± 0.008	0.058 ± 0.010^1^	0.061 ± 0.010^1^	0.061 ± 0.004
Epididymides	1.571 ± 0.123	1.596 ± 0.188	1.663 ± 0.219	1.739 ± 0.137
Thymus	0.314 ± 0.045	0.358 ± 0.061	0.322 ± 0.049	0.294 ± 0.080
Females				
Brain	1.79 ± 0.14	1.88 ± 0.07	1.92 ± 0.13	1.88 ± 0.07
Heart	0.93 ± 0.09	0.97 ± 0.08	0.96 ± 0.07	0.93 ± 0.06
Kidneys (paired)	1.84 ± 0.09	1.86 ± 0.18	1.85 ± 0.13	1.78 ± 0.10
Liver	6.34 ± 0.58	6.70 ± 0.84	6.51 ± 0.40	6.50 ± 0.38
Spleen	0.62 ± 0.09	0.66 ± 0.13	0.64 ± 0.07	0.58 ± 0.07
Uterus	0.65 ± 0.16	0.77 ± 0.23	0.67 ± 0.19	0.66 ± 0.18
Adrenals (paired)	0.069 ± 0.009	0.071 ± 0.006	0.073 ± 0.010	0.073 ± 0.009
Ovaries	0.118 ± 0.024	0.129 ± 0.029	0.140 ± 0.048	0.135 ± 0.042
Thymus	0.219 ± 0.041^1^	0.250 ± 0.061	0.254 ± 0.049	0.253 ± 0.054

^1^Mean and standard deviation for 9 animals

**Table 6:** Mean organ-to-brain weight ratios of male and female rats that consumed PGX^® ^for 90 days

Organ	Control	1.25%	2.5%	5.0%
Males				
Heart	0.72 ± 0.06	0.74 ± 0.04	0.71 ± 0.05	0.72 ± 0.07
Kidneys (paired)	1.56 ± 0.11	1.47 ± 0.09	1.47 ± 0.17	1.45 ± 0.09
Liver	5.63 ± 0.58	5.64 ± 0.43	5.87 ± 0.96	5.90 ± 0.37
Spleen	0.41 ± 0.07	0.38 ± 0.04	0.37 ± 0.03	0.40 ± 0.03
Testes (paired)	2.04 ± 0.12	2.05 ± 0.12	2.02 ± 0.18	2.05 ± 0.15
Adrenals (paired)	0.032 ± 0.005	0.030 ± 0.005	0.031 ± 0.006	0.031 ± 0.003
Epididymides	0.795 ± 0.056	0.810 ± 0.090	0.833 ± 0.86	0.873 ± 0.073
Thymus	0.160 ± 0.029	0.181 ± 0.029	0.161 ± 0.017	0.148 ± 0.042
Females				
Heart	0.52 ± 0.06	0.52 ± 0.05	0.50 ± 0.03	0.49 ± 0.04
Kidneys (paired)	1.03 ± 0.06	0.99 ± 0.08	0.97 ± 0.11	0.95 ± 0.06
Liver	3.56 ± 0.39	3.57 ± 0.39	3.42 ± 0.34	3.47 ± 0.26
Spleen	0.35 ± 0.06	0.35 ± 0.06	0.34 ± 0.05	0.31 ± 0.03
Uterus	0.37 ± 0.09	0.41 ± 0.13	0.35 ± 0.10	0.35 ± 0.09
Adrenals (paired)	0.039 ± 0.007	0.038 ± 0.003	0.039 ± 0.007	0.039 ± 0.005
Ovaries	0.066 ± 0.014	0.069 ± 0.015	0.074 ± 0.029	0.072 ± 0.021
Thymus	0.124 ± 0.025	0.133 ± 0.032	0.134 ± 0.028	0.135 ± 0.028

## Discussion

The composition, solubility, and viscosity of fibrous products classified as "nondigestible" may have various effects on the absorption, metabolism, and excretion of ingested material. Brown *et al*. [2] found that various soluble fibers (*e.g*., oat, psyllium, or pectin fiber) reduce total and LDL serum cholesterol in clinical studies, but did not affect triacylglycerol and HDL cholesterol levels. Nondigestible fibers are resistant to the action of salivary, intestinal, and pancreatic hydrolases, but may be readily fermented by bacteria in the cecum/large intestine, hydrolyzing to short chain fatty acids (SCFA), thus releasing hydrogen and other gases [1]. Several SCFA have been shown to have anti-inflammatory properties and may have a beneficial effect on inflammatory bowel disease in culture [15,16]. Viscous fibers have been found to alter blood glucose and cholesterol concentrations, as well as prolonging gastric emptying time and slowing transit time through the small intestine [17-19]. Increased consumption of dietary viscous fiber has also been shown to reduce serum glucose increases during meal consumption, decrease plasma cholesterol, and improve the colonic ecology in clinical trials [9] and modify the absorption of fats, minerals and bile acids as well as influence appetite and absorb toxins [20-23].

PGX is manufactured from three different viscous dietary fibers by a proprietary process: one fermentable β-glycan fiber, an alginate, and a polysaccharide gum. Indigestible carbohydrates such as alginates and gums are not digested in the small intestine, but pass into the large intestine where the bacterial flora ferment these fibers (β-glycan to a greater degree), resulting in the release of SCFA [9,24]. SCFA may be absorbed in the colon and help to provide a reduced intake of calories and could result in a subsequent prolongation of satiety [10,25,26]. Jenkins *et al. *[23,27]. reported that the consumption of viscous fiber may also slow gastric emptying and small intestinal absorption of carbohydrates, resulting in reduced postprandial insulin secretion as well as lower LDL cholesterol and apolipoprotein B concentrations. Research has found that consumption of glucomannan and sodium alginate decreases serum total, HDL and LDL cholesterol in human trials [28,29]; however, it was determined that these changes are modulated by increased fecal steroid excretion and not increased cholesterol excretion [22]. Interestingly, in the present study, PGX increased aminotransferase levels in female rats, although glucomannan has been shown to reduce increased aminotransferase levels in rats fed a high cysteine diet [30]. Conversely, the male rats that consumed 5.0% PGX showed a nonsignificant decrease in these enzyme levels. The changes in aspartate (AST) and alanine (ALT) aminotransferase enzyme levels found in the female rats did not correlate with either histopathological changes to the liver morphology or absolute or relative organ weight changes, nor were the values double that of control, which is one indicator of biological significance [31]. These low levels of changes may be the result of an adaptive process to the consumption of this viscous polysaccharide at the 5.0% level of intake, and may be due to increased protein turnover [32]. Neither of these enzymes are liver-specific in rats. Both enzymes are also found in skeletal and cardiac muscle [33]. However, no skeletal or cardiac muscle changes (histological or organ weight) were found in any of the animals in the present study. Further, no other correlating liver enzyme levels were affected. Sorbitol dehydrogenase is liver-specific in rats, but this liver enzyme was not affected in either the males or females. In addition, alkaline phosphatase, a membrane-bound enzyme with the highest activity in osteoblasts, biliary epithelium and epithelial cells of the kidney and intestines was not affected. The statistically significant changes in AST and ALT were noted to reside just outside the tight standard deviations, and thus appear to be of minimal clinical significance. Therefore, these findings were not considered toxicologically significant. The increase in triglyceride concentrations in the females fed 5.0% PGX, although unexpected, was not a part of a dose-response effect, and did not correlate with an increase in cholesterol levels. Decreased red blood cell count occurred in the high dose males, while significant decreases in serum sodium, potassium and chloride concentrations were observed in the females fed 5.0% PGX. However, the decreased mineral concentrations may be the result of significantly increased urinary volume in both males and females at the high dose, with a concomitant decrease in urinary specific gravity (males and females) and protein concentration (females). The diet was provided in powder form, and the high oral viscosity of PGX may have increased water intake, thereby resulting in the lower red blood cell count and lower plasma mineral levels. These results were within historical control values, did not correlate with any histopathological changes, and were not considered adverse.

PGX is a highly purified, water soluble, viscous polysaccharide product. The overall benefits and physiologic effects of a soluble polysaccharide to human health are directly proportional to its structural, chemical and physical properties [20], specifically, viscosity [23]. Viscous gums increase stool volume and bulk, partially through increased water retention and adsorption of organic materials [18,21]. Eastwood *et al*. [34] found that high levels of xanthan gum administered to men (*n *= 5) for 23 days had no significant effect on many parameters, including plasma biochemistry, hematology, urinalysis parameters, glucose, triglycerides, phospholipids, or HDL cholesterol, although daily fecal bile was significantly increased. A recent clinical trial found that PGX administered to subjects for 21 days did not result in adverse effects, but did decrease serum cholesterol [35]. The highest treatment dose in this current study found decreased serum sodium, potassium, and chloride levels in the female rats. These effects may be related to the increased urine volume observed, which could also account for the increase the urine pH and decrease the specific gravity found in both the male and female rats. Similar, though more excessive, urine changes were noted in mice that consumed high levels of sodium alginate (25% of the diet) for 89 weeks, and were found to be reversible upon cessation of the diet [36]. The changes in the high dose group were within historical control values, and the low variability between the animals may have contributed to the statistical significance of these changes.

The observed effects of this study have been reported in previous high dose studies of similar substances in rats, and can be explained in terms of the predictable physiological adaptive response to a high level of both indigestible and fermentable dietary fibers [21,36,37]. In the present study, no differences in any organ tissue under histopathological analysis were observed between control and treated animals, supporting the hypothesis that the observed minimal clinical changes were adaptive in nature and/or a result of the increased urinary volume.

## Conclusion

In summary, administration of PGX to rats at up to 5.0% of their diet (3219 and 3799 mg/kg bw/day in male and female rats, respectively) for 90 days produced several urinalysis and serum chemistry changes that were not correlated histopathologically. These changes likely represent a nutritional effect as the result of dietary displacement at the high dose, and were not considered toxicological in nature. Therefore, a NOAEL for PGX is at 5.0% of the diet, corresponding to 3219 and 3799 mg/kg bw/day in male and female rats, respectively.

## Competing interests

All authors have a financial relationship with the sponsor of the study, InovoBiologic, Inc., Calgary, Alberta, Canada.

## Authors' contributions

*These authors contributed equally to this work. RAM participated in the design of the study, analyzed the results and drafted the manuscript. MRL participated in the analysis of the results and composition of the manuscript. SW initiated the study, participated in its design, analysis of the results, composition of the manuscript and coordination. PAM participated in the design of the study, performed the study assays, data analysis and manuscript editing. DJM participated in the design of the study, performed the study assays, and analyzed the results. GAB participated in the design of the study and contributed to the composition of the manuscript. All authors read and approved the final manuscript.
